# The Pharmacist’s Role in the Implementation of FASTHUG-MAIDENS, a Mnemonic to Facilitate the Pharmacotherapy Assessment of Critically Ill Patients: A Cross-Sectional Study

**DOI:** 10.3390/pharmacy10040074

**Published:** 2022-06-30

**Authors:** Jose Miguel Chaverri-Fernández, Esteban Zavaleta-Monestel, Josué Murillo-Cubero, José Pablo Díaz-Madriz, Brayan Leiva-Montero, Sebastián Arguedas-Chacón, Raquel Arguedas-Herrera

**Affiliations:** 1 Department of Pharmacology, Toxicology and Pharmacodependence, University of Costa Rica, San José 11501, Costa Rica; jose.chaverri@ucr.ac.cr; 2 Department of Pharmacy, Hospital Clínica Bíblica, San José 10104, Costa Rica; josuem@gmail.com (J.M.-C.); jdiazm@clinicabiblica.com (J.P.D.-M.); 3 Pharmacy Intern, Hospital Clínica Bíblica, San José 10104, Costa Rica; brayanjlm@gmail.com (B.L.-M.); s.arguedasch@gmail.com (S.A.-C.); raq.ah1127@gmail.com (R.A.-H.)

**Keywords:** critical care, pharmacotherapy, drug-related problems, delirium, interventions

## Abstract

FASTHUG is a mnemonic used by intensive care physicians to ensure the proper management of patients admitted to an Intensive Care Unit (ICU). FASTHUG-MAIDENS is a modified version that incorporates key pharmacotherapeutic elements such as delirium management, drug dosing, and drug interactions for an appropriate medication assessment of critically ill patients. An analytical cross-sectional study of hospitalized patients was carried out to determine aspects related to the pharmacotherapeutic management of critically ill patients that required to be optimized, to design and implement a protocol based on the FASTHUG-MAIDENS mnemonic. A total of 435 evaluations were performed to assess the status of current critical patient management. The main parameters with opportunities to be improved were analgesia, feeding, and sedation. With the implementation of MAIDENS, the parameters of analgesia, sedation, and thromboprophylaxis showed an increase in the percentage of optimal management. Furthermore, 103 drug-related problems were detected, and most of them were associated with feeding (21.3%), glucose control (11.7%), and delirium (9.7%). The FASTHUG MAIDENS protocol implementation allows for the evaluation of more vital aspects in the management of critically ill patients. The daily review of patients admitted to the ICU by a clinical pharmacist (CP) using the FASTHUG-MAIDENS checklist instead of the FASTHUG mnemonic facilitates the identification of DRPs for the performance of possible interventions by the CP to improve the pharmacotherapeutic management.

## 1. Introduction

FASTHUG is a mnemonic created by intensivists to facilitate the management of critically ill patients in the Intensive Care Unit (ICU). It considers key clinical elements such as Feeding, Analgesia, Sedation, Thromboembolic prophylaxis, Head-of-bed elevation, stress Ulcer prophylaxis, and Glucose control [1]. As clinical practice advances and other healthcare professionals have been able to provide more support to critically ill patients, several modifications of the mnemonic were created to cover the specific needs of each ICU. One of those modifications was FASTHUG-MAIDENS, which was designed to help pharmacists during the daily monitorization of pharmacotherapeutic aspects in critically ill patients [2].

FASTHUG-MAIDENS keeps basic aspects of the original mnemonic (except Head-of-bed elevation, which is included in Feeding) and incorporates different factors related to pharmacotherapy assessment such as the evaluation of drug dosing, drug indications, dose adjustments in kidney and liver failure, drug interactions, medication duplicities, drug allergies, an adequate medication reconciliation, the promotion of the rational use of antibiotics, and the assessment of the effectiveness and security of the drugs used [2]. Some studies showed that the use of a protocol based on the FASTHUG-MAIDENS mnemonic increased the detection of drug-related problems (DRP) in an ICU, which can have a direct impact on the patient’s health in terms of healthcare cost, length of stay (LOS), and even mortality [3,4,5].

Pharmacists may play an important role in an ICU, helping to prevent, detect, assess, and provide interventions to reduce the impact of DRP. Most of these problems are related to polypharmacy, medication reconciliation process errors, patient comorbidities, and the need for dosing adjustment in kidney and liver failure [6]. Furthermore, the presence of a pharmacist in an ICU has shown several benefits, such as a decrease in the duration of mechanical ventilation and length of stay (LOS) in the ICU, the implementation of a sedation protocol, a reduction in the costs of healthcare in critically ill patients with infections [7], and even an increase in the detection of drug interactions that resulted in a reduction of mortality [8].

This study aims to assess the benefits of the use of FASTHUG-MAIDENS as a standardized pharmacy approach to facilitate the daily pharmacotherapy evaluation and monitorization of critically ill patients and, thus, support the contribution of a clinical pharmacist in the ICU.

## 2. Materials and Methods

### 2.1. Study Design and Settings

This is an observational cross-sectional study of hospitalized, critically ill individuals involving patients admitted to the ICU of the Clínica Bíblica Hospital (HCB), a 78-bed private health center located in San José, Costa Rica. The retrospective analysis was made between July 2018 and June 2019, and the prospective analysis was made between February and May 2021.

### 2.2. Inclusion and Exclusion Criteria

Patients who were hospitalized during the defined periods were identified via the electronic clinical records of the hospital, and all the patients admitted to the ICU were included. Patients under 18 years of age, with a LOS in the ICU of less than two days, with a terminal illness or in palliative care, and with a positive diagnosis for COVID-19 were excluded.

### 2.3. Definitions

For the monitorization process, the parameters of the mnemonic were defined as follows:

- Feeding: to determine the type of diet that best suits the characteristics of each patient, the algorithm proposed by Doig et al. was used [9]. In general, the following aspects were evaluated for every patient who received enteral/parental nutrition: the presence of drug-food interactions, protein intake between 1.2–2.0 g/kg (real body weight), daily caloric requirements between 25–30 kcal/kg/day, and an angle of the head of the bed of 30–45° in patients with enteral nutrition [9].
- Analgesia: to determine the level of pain, the Behavioral Pain Scale (BPS) was used, considering a score greater than 5 as significant pain [10]. The Visual Analog Pain Scale (VAS) was used when the patient’s condition allowed it [11]. In addition, opioid dose reductions via the use of adjuvants and significant adverse effects were monitored before performing a painful medical procedure [12].
- Sedation: the Richmond Agitation-Sedation Scale (RASS) was used [13], considering the risk-benefit of each prescribed medication and the clinical characteristics of each patient [12].
- Thromboprophylaxis: the clinical guidelines of the HCB and the international treatment guidelines were used. The Padua Scale and the Caprini Risk Analysis Model were used to determine the need for thromboprophylaxis, and the suitability, dose, and route of administration of the selected drug were evaluated, as well as the patient’s contraindications to the use of the medication [14].
- Hyperactive or hypoactive delirium: for the daily monitoring of delirium risk in patients with a RASS score between −2 and 0, the Confusion Assessment Method for the ICU (CAM-ICU) was used. Based on international guidelines, the use of drugs to prevent delirium and the use of pharmacological and non-pharmacological measures for the treatment of delirium were evaluated [15].
- Stress ulcer prophylaxis: the use of medications for this parameter was evaluated in patients with risk factors such as respiratory distress (mechanical ventilation > 48 h), coagulopathy (platelets < 50,000/mm^3^, INR > 1.5 or TTPa twice the base value) and hepatic or renal disease, among others. The evaluation included doses, route of administration, and adverse effects of medication, especially those of great clinical relevance, such as C. difficile infection or pneumonia.
- Glucose control: the blood glucose level and hypoglycemic medications were reviewed. In addition, compliance with other aspects such as maintenance of glucose level between 140–180 mg/dL, management of hypoglycemia (glucose level < 70 mg/dL), and use of subcutaneous insulin were assessed.
- Medication reconciliation: chronic treatments of the patients were reviewed on the day of admission to the ICU. An evaluation of the need to maintain chronic pathologies under control was made, under the management of the acute condition of the patient (drug interactions and disease impediments).
- Antibiotics or antimicrobial agents: there was a review of whether drug selection, dose, route of administration, and frequency were in the diagnosis and signs and symptoms of the patients. In addition, dose adjustment and de-escalation after the results of cultures were reviewed. For this analysis, the Sanford Guide tool, the IDSA guidelines, and the HCB’s pharmacoepidemiologic reports were used [16,17].
- Indications for medications: it was checked that all medications and their prescribed doses and route of administration had an appropriate indication, using the UpToDate database [18].
- Drug dosing: drug doses were reviewed following the patient’s diagnosis and symptoms. For dose adjustment, the glomerular filtration rate was used as an indicator of renal function, and the Child−Pugh score was used as the liver function parameter.
- Electrolytes, hematology, and other laboratory results: daily revisions of these parameters were made via the electronic hospital records.
- No drug interactions, allergies, duplications, or side effects: the medical history of the patients was reviewed to determine if medications were prescribed for which the patient had previously reported an allergy if there was no duplication of therapy and if side effects were related to the treatment and drug interactions. For drug interactions, UpToDate’s Lexi-Comp^®^ Drug Interactions tool was used.
- Stop dates: the indications given by the treating physicians regarding the duration of treatment were reviewed. In addition, it was verified that the duration of treatment was in accordance with the indication.

### 2.4. Data Collection

The study was divided into two stages. Initially, a retrospective evaluation was performed using the FASTHUG mnemonic. In this period, the data was re-collected using electronic clinical records. The aspects assessed for each parameter of the mnemonic were selected using the corresponding international treatment guidelines and the information provided in the original FASTHUG article by Vincent [1]. After data collection, an analysis was carried out and used as a diagnostic tool to determine which aspects related to the management of critically ill patients needed to be optimized.

Subsequently, the FASTHUG-MAIDENS protocol was created and implemented, which was carried out prospectively with daily reviews of patients admitted to the ICU. For the development of this protocol, key pharmacotherapeutic aspects were considered for each variable of the mnemonic. Reviews were performed daily by the clinical pharmacist (CP) in the ICU, using a spreadsheet in Microsoft^®^ Excel developed by the pharmacy department containing all the parameters mentioned above, to evaluate each patient.

Upon finding any discrepancy or limitation related to the parameters, interventions were carried out through the CP when it was considered clinically relevant. Drug-related problems and interventions were documented and stratified using Cypolle’s DRP classification system, and every intervention was followed up to assess the possible benefit of treating the patient [19]. The interventions were made as suggestions directly in person to the treating physician. The pharmaceutical interventions were recorded in the electronic clinical records and categorized as accepted, partially accepted, and rejected in the clinical records.

Finally, the results were analyzed and used as evidence for the incorporation of actions into the current hospital’s clinical guidelines, to optimize the therapy of critically ill patients.

### 2.5. Statistical Analysis

Data was entered, classified, and analyzed using Microsoft^®^ Excel 2019 (Microsoft, Redmond, Washington, DC, USA) and IBM^®^ SPSS Statistics version 28 (IBM, Chicago, IL, USA). Descriptive statistics were conducted such as frequencies and percentages. The percentage of optimal management for each variable was established according to the international treatment guidelines, the original articles for each mnemonic, and the protocol created.

### 2.6. Ethics Approval and Consent to Participate

Ethical approval to conduct the study was obtained from the Scientific Ethical Committee of the University of Costa Rica, Costa Rica (approval date 9 June 2021), approval reference number CEC-374-2021. Written consent was not necessary for this study because the direct interventions were with the treating physicians.

## 3. Results

### 3.1. Patient Demographics

A total of 140 patients met the inclusion criteria for the present study, 120 in the assessment stage (Stage 1) and 20 in the stage after the implementation of the protocol (Stage 2), and their demographics and admission diagnosis are shown in Table 1. A total of 528 evaluations were performed, 435 in the first stage and 93 in the second stage.

### 3.2. Assessment Stage

**Figure 1:**
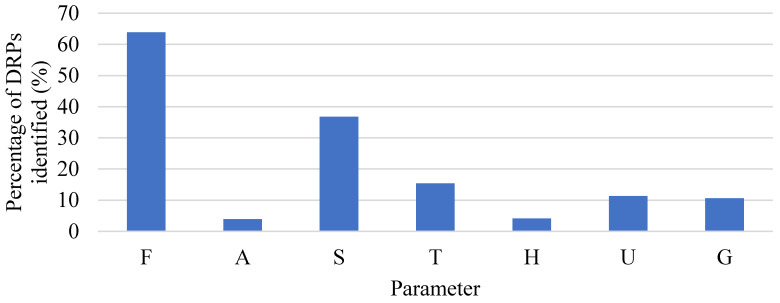
Distribution of drug-related problems (DRPs) identified using the FASTHUG mnemonic in the first stage in 120 patients, with each category expressed as a total (n=435). F = Feeding; A = Analgesia; S = Sedation; T = Thromboembolic prophylaxis; H = Hypoactive or Hyperactive delirium; U = stress Ulcer prophylaxis; G = Glucose control.

Several opportunities for improvement were found, mainly related to the parameters of Feeding, Analgesia, and Sedation; additionally, these parameters were the ones with the highest amount of DRPs (Figure 1). For Feeding, in 51.0% of the evaluations the patients received a daily caloric intake below the recommendation, in 5.3% the patients received an intake over the recommendation, and in only 14.9% did the patients receive an adequate daily caloric intake. Finally, in 28.8% of the evaluations, the daily caloric intake was not quantifiable due to food restriction before a surgical procedure or oral nutrition.

For Analgesia and Sedation, most of the DRPs were related to the lack of an adequate process for the monitorization of pain and sedation levels; in 43.6% of evaluations, a validated tool was not used to estimate the pain level, and in 62.3% of the cases, the sedation level was not calculated or a non-validated tool was used. Furthermore, several drug-related problems were detected, mainly associated with drug dosing, adverse drug reaction, and unnecessary drug therapy.

### 3.3. Implementation of the Protocol

**Figure 2:**
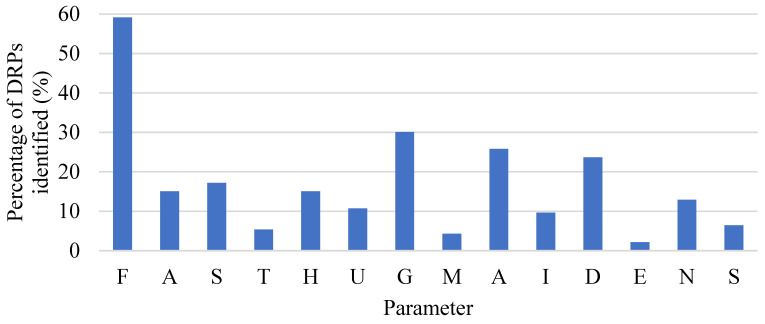
Distribution of drug-related problems (DRPs) identified using the FASTHUG-MAIDENS mnemonic in the second stage in 20 patients, with each category expressed as a total (n=93). F = Feeding; A = Analgesia; S = Sedation; T = Thromboembolic prophylaxis; H = Hypoactive or Hyperactive delirium; U = stress Ulcer prophylaxis; G = Glucose control; M = Medication reconciliation; A = Antibiotics or Anti-infectives; I = Indications for medications; D = Drug dosing; E = Electrolytes, hematology, and other laboratory tests; N = No drug interactions, allergies, duplication, or side effects; S = Stop dates.

A reduction in the percentage of DRPs for the parameters of Analgesia, Sedation, Thromboprophylaxis, and ulcer Stress prophylaxis was achieved during the second stage, while several DRPs that required improvement were found in the MAIDENS mnemonic (Figure 2).

**Figure 3:**
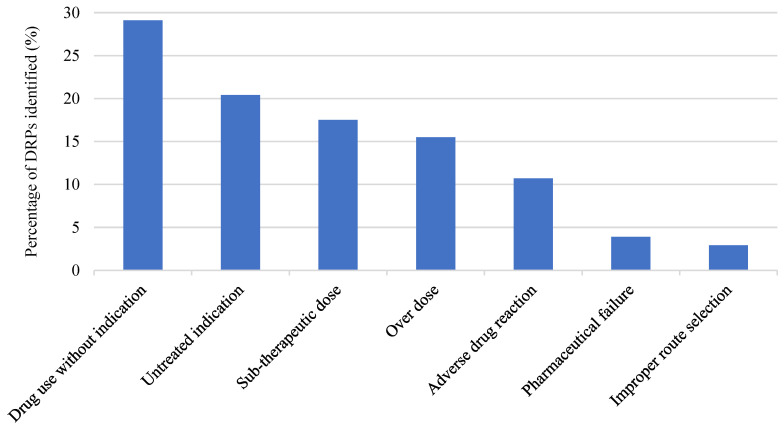
Distribution of drug-related problems (DRPs) identified, according to the Hepler–Strand classification, for 20 patient encounters, with each category expressed as a percentage of total DRPs identified (*n* = 103).

An average of 5.15 DRPs was detected for each patient monitored with the protocol, with a total of 103. Those problems were classified using the Hepler–Strand DRP classification system, adding a new category for pharmaceutical failure and improper route selection, as shown in Figure 3. Most of the DRPs were related to the parameter of Feeding (*n* = 22), Glucose control (*n* = 12), and Hyper or Hypoactive delirium (*n* = 10).

**Figure 4:**
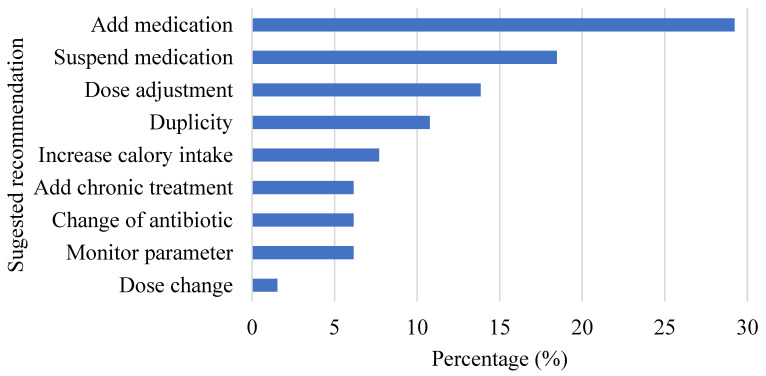
Distribution of pharmaceutical interventions provided by the clinical pharmacist in the intensive care unit for 20 patient encounters, with each category expressed as a percentage of total DRPs identified (*n* = 65).

**Figure 5:**
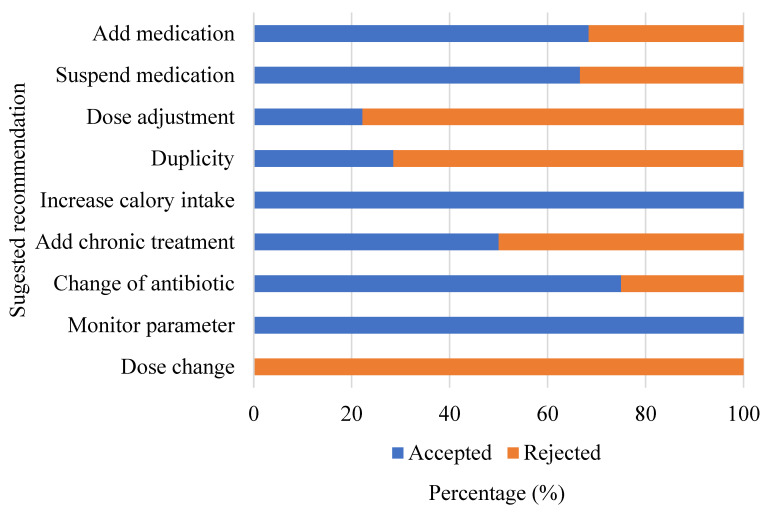
Acceptance distribution of pharmaceutical interventions provided by the clinical pharmacist in the intensive care unit for 20 patient encounters, with each category expressed as the total (*n* = 65).

As shown in Figure 4, after the implementation of the protocol, a total of 65 interventions were carried out, with an average of 3.25 interventions per patient. Most of the interventions were performed in the parameters of Antibiotics and Anti-infectives (*n* = 12), Feeding (*n* = 9), ulcer Stress prophylaxis (*n* = 7), and drug dosing (*n* = 7). An acceptance rate of 60% was accomplished, and the accepted interventions (*n* = 39) promoted changes in the drug therapy of the patients, which allowed the management of the patients to follow the treatment guidelines (Figure 5).

In contrast, in the rejected interventions group (*n* = 26), the pharmacotherapeutic management remained far from what is recommended in the guidelines. Despite this, it was not possible to determine whether this influenced the clinical condition of the patient.

## 4. Discussion

This study was conducted to evaluate the importance of CP participation in a multidisciplinary ICU team. Pharmaceutical interventions are a very important process in evaluating the patient’s management and in providing patient-centered care, making recommendations, and preventing or resolving DRPs [20]. Before the CP submits a proposal, an evaluation of the patient’s overall clinical status is necessary. The development of standardized protocols facilitates the decision-making process and the daily monitoring in the ICU. For instance, the use of the FASTHUG mnemonic has shown a decrease in ventilator-associated pneumonia and mortality in the ICU [20,21,22].

During the first stage of the study, we conducted a retrospective analysis using the FASTHUG mnemotechnic; meanwhile, in the second stage, in addition to the application of the FASTHUG-MAIDENS spreadsheet, patients admitted into the ICU were given daily visits by the CP, allowing for more personalized monitoring of these patients.

The reason for having a smaller sample size for the second stage of the study is that during that period, the country was facing a critical moment in the COVID pandemic, and in general, the number of non-COVID ICU patients decreased drastically. Additionally, because of access limitations, due to the isolation of the COVID-ICU beds, it was not possible to perform the daily visits required to carry out monitoring using this mnemonic. Therefore, the decision was made to exclude these patients.

In both stages of this study, the parameter of Feeding had non-optimal management, mostly due to the administration of a low-calorie, low-protein diet, which has been associated with an increase in complications such as acute respiratory distress syndrome (ARDS), infections, kidney failure, pressure ulcers, and increased mortality [23].

The CP plays a crucial role within the ICU multidisciplinary team, evaluating specific requirements of the patients to design a nutritional formulation. Furthermore, a CP can monitor the patient’s response to the preparation and suggest specific changes if applicable [23]. The presence of a CP within the multidisciplinary team of an ICU produced an increase in the number of patients who reached the daily goals of protein and calorie intake, which decreased hospitalization time and mortality [24].

In the present study, an average of 5.5 medication-related problems were found for each patient admitted to the ICU. This result is consistent with the findings obtained in a systematic review carried out in 2013, where the number of DRPs per patient found by a CP ranged from 0.13 to 10.6 [25]. The presence of a CP within the ICU improves the ability to optimize pharmacological therapy, prevents the appearance of these DRPs, and minimizes the effects they may have on the patient’s clinical condition.

After the implementation of the FASTHUG-MAIDENS protocol, some of the percentages of DRPs decreased. In other parameters, such as Analgesia, Hyperactive or Hypoactive delirium, and Glucose control, the percentages increased notably. The increase in these percentages was an opportunity for the CP to intervene in the patient’s therapy. These kinds of interventions have been linked to a decrease in the LOS in the ICU and mortality [26].

Regarding the interventions, a percentage of acceptance of 60.0% was obtained, which is consistent with the findings obtained in another study, with an acceptance percentage for interventions carried out by pharmacists ranging between 39.0% and 100.0% [25]. Recommendations with the highest percentage of acceptance were an increase in calorie intake (100%), monitorization of parameters (100%), and change of antibiotic (75%).

The acceptance rate for the interventions can be used as an indicator of their clinical relevance. Additionally, it can help to detect the need to improve multidisciplinary collaboration for the optimization of the therapy [27]. In some cases, direct verbal communication and close collaboration between the members of the multidisciplinary team in the ICU increase the rate of interventions accepted by the treating physician, thus also increasing the impact that the CP has on the pharmacological treatment of patients [28,29].

In this study, the suggested recommendations with the highest rejection percentages were dose change, dose adjustment, and duplicity. Approximately 36.4% of patients with recommendations of dose change and dose adjustment had renal insufficiency. These patients often require a reduction in drug doses because of their renal dysfunction, and in most cases, the treating physician considered the dose change unnecessary because of the general state of the patient. For chronic treatment, the interventions made by the CP highlighted the importance of medication reconciliation, which is important for treatment safety and the reduction of medical costs [20,30].

## 5. Conclusions

The FASTHUG and FASTHUG MAIDENS mnemotechnics are useful tools for monitoring critically ill patients. In this study, we determine that the FASTHUG MAIDENS mnemotechnic presents the advantage of allowing the clinical pharmacist to evaluate vital aspects of the management of ICU patients and facilitates the identification of DRPs. The use of this type of protocol allows pharmacists to be more active members of multidisciplinary teams responsible for ICU patients. Additional research on this topic to assess the impact of this mnemotechnic in the improvement.

## Figures and Tables

**Table 1:** Baseline demographic and clinical characteristics.

Characteristics	Stage 1 (*n* = 120)	Stage 2 (*n* = 19)
Demographics		
Age (years), median (IQR)	71 (19–100)	69 (30–91)
Respiratory support		
Mechanical Ventilation, *n* (%)	30 (25.0)	8 (42.1)
Admission diagnosis, *n* (%)		
Cardiovascular disorders ^1^, *n* (%)	45 (37.5)	7 (35.0)
Respiratory disorders ^2^, *n* (%)	27 (22.5)	4 (20.0)
Neurologic disorders ^3^, *n* (%)	13 (10.8)	-
Gastrointestinal disorders ^4^, *n* (%)	11 (9.2)	-
Sepsis, *n* (%)	3 (2.5)	1 (5.0)
Trauma ^5^, *n* (%)	6 (5.0)	1 (5.0)
Metabolic disorders ^6^, *n* (%)	5 (4.2)	1 (5.0)
Convalescence phase due to COVID-19	-	4 (20.0)
Other ^7^, *n* (%)	10 (8.3)	1 (5.0)
Pharmacy interventions	-	63
Daily interventions, median (IQR)	-	0.65 (0–4)

Abbreviation: IQR, interquartile range. ^1^ Arrhythmia, catheterization, angiography, angioplasty, cardiac ablation, ischemic heart disease, pacemaker placement, stent placement, hypertensive crisis, heart attack, heart failure, acute coronary syndrome, thrombolysis, deep venous thrombosis, and superior vena cava syndrome. ^2^ Pleural effusion, respiratory distress, pneumonia, pneumothorax, Chronic Obstructive Pulmonary Disease Exacerbation, and pulmonary embolism. ^3^ Pleural effusion, respiratory distress, pneumonia, pneumothorax, Chronic Obstructive Pulmonary Disease Exacerbation, and pulmonary embolism. ^4^ Cholecystitis, acute diverticulitis, esophagus necrosis, obstructive colon neoplasm, intestinal occlusion, and digestive bleeding. ^5^ Brain contusion, scalp detachment, hip fracture, vertebral dislocation, and polytrauma. ^6^ Diabetes decompensation, electrolytic disorder. ^7^ Functional Endoscopic Sinus Surgery (FESS), prostatic artery embolization, acute kidney disease, left partial nephrectomy, Stevens-Johnson syndrome.

## Data Availability

The data presented in this study are available on request from the corresponding author.
